# Codesign and community outreach to create COVID-19 safe communities: A Karen community case study

**DOI:** 10.3389/fpubh.2023.1081767

**Published:** 2023-03-24

**Authors:** Hilary Davis, Shandell Elmer, Kaye Graves, Caitlin Learmonth

**Affiliations:** ^1^The Centre for Social Impact, Faculty of Business and Law, Swinburne University of Technology, Melbourne, VIC, Australia; ^2^The Centre for Global Health and Equity, School of Health Sciences, Swinburne University of Technology, Melbourne, VIC, Australia; ^3^Bendigo Community Health Services, Bendigo, VIC, Australia

**Keywords:** outreach, codesign, COVID-19, Karen community, vaccination uptake, case study, health literacy, place-based research

## Abstract

During the COVID-19 pandemic, government directives for health and community services focused on building capacity for COVID-19 safe behaviors. During 2020–2021, there was mounting pressure to increase vaccination numbers to boost population-wide immunity, thereby enabling the lessening of pandemic response restrictions. The Australian population, in general, faced communication hurdles in understanding COVID-19, government directives and policies, and health initiatives. This was particularly challenging given the rapid changes in disease behaviors and community response requirements. This community case study documents local experience in delivering information about COVID-19 safety and vaccination to a former refugee community (the Karen community) in regional Victoria. Community outreach and codesign approaches established closer engagement between the Karen community and Bendigo Community Health Services (BCHS). This case study is explored through semi-structured interviews conducted face-to-face and *via* videoconferencing with key Karen community leaders, Karen community members, vaccination clinic volunteers, and BCHS staff and bicultural workers. A hybrid approach that employed community outreach and codesign approaches in tandem built trust and closer ties between the Karen community and BCHS, leading to increased understanding and compliance with COVID-19 safe messages and vaccination uptake. Community-led innovations included codesign of COVID-19 fact sheets and videos in the Karen language, involvement of “local champions,” assisting Karen businesses with COVID-19 safe plans, and creation of a COVID-19 information hotline. The latter was facilitated by BCHS bicultural staff. These innovations supported the delivery of vaccination clinics at the local Karen Temple. Embedding multi-level, tailored, and responsive public health approaches is particularly important in complex settings where there are disproportionately high levels of community disadvantage, as occurred during the COVID-19 pandemic.

## 1. Introduction

The coronavirus disease 2019 (COVID-19) pandemic created global health challenges, interrupting public health agendas, and necessitating the reconsideration of the ways healthcare services and interventions are delivered. The COVID-19 pandemic gave rise to unprecedented public health responses in Australia. A suite of interventions included early closure of international and state borders, mandatory home isolation for travelers, and tracking of cases while encouraging vaccine uptake. The public health orders and restrictions in response to COVID-19 were most severe in Melbourne, the state capital of Victoria, which reported the highest number of cases, hospitalizations, and deaths of any Australian state or territory up to December 2021. Melbourne reported the longest lockdowns of any city in the world (as of October 2021), enduring cumulatively 262 days of lockdowns across six time periods in response to COVID-19 infection outbreaks (1).

Health and community services evolved their focus on building capacity for COVID-19 safe behaviors in response to federal and state government directives over the trajectory of the pandemic. However, these public health emergency directives and restrictions left many people feeling threatened, insecure, and fearful. People who were already experiencing vulnerability and disadvantage were disproportionately impacted by the pandemic strain (2). During 2021, the emphasis was on implementing the federal government’s COVID-19 vaccine rollout strategy and increasing vaccination uptake, thereby enabling the lessening of public health restrictions including border closures, in line with federal/state guidelines or roadmaps (3). As vaccination rates began to stall in mid-2021, particular attention was placed on communities with low rates of vaccination such as people without English as a main language, migrant or former refugee communities, people with health literacy needs and challenges, and people living in crowded or insecure housing conditions (4). People in these communities were being left behind as the messages about COVID-19 safety, and vaccinations did not reach them as the policies were designed and implemented without due regard for the inequalities and vulnerabilities of key communities (5).

This article outlines a community case study of the approaches used by the Bendigo Community Health Services (BCHS) to partner with a community at risk of bearing a greater pandemic burden than others, the local Karen community, by building their COVID-19 safe capacity and behaviors. The Karen community is indigenous to the Thailand–Burma border region in Southeast Asia and one of many ethnic groups in Myanmar (formerly Burma). It is estimated that over 4,000 Karen refugees have re-settled in Bendigo in the last 12 years, making this one of the largest Karen communities in Australia. The Karen community in Australia experiences disadvantages as in their home country they have not had access to universal health and education, including disease prevention/screening programs and immunizations. Furthermore, past experiences of trauma may present as fear of authority figures including health and community services (e.g., doctors, paramedics, police officers, and emergency services). This case study draws upon interviews with Karen community members and key leaders, vaccination clinic volunteers, and BCHS staff and bicultural workers that were conducted face-to-face and *via* videoconferencing in late 2021 and early 2022.

## 2. Community and health service context

### 2.1. Bendigo Community Health Services

The BCHS is in the City of Greater Bendigo in the state of Victoria. Bendigo has a population of approximately 121,000 and is situated approximately 150 km northwest of Melbourne, the state capital. BCHS is a not-for-profit organization that provides a range of primary and community health services free of charge or at a minimal cost. BCHS has a team of experienced healthcare professionals and works in partnership with other health services across central Victoria, and with local community members. It provides a wide range of services, including counseling services, alcohol and drug group programs, pediatric services, community education, and preventative and intervention services. BCHS employs a team of refugee project workers, which includes 12 bicultural staff who are members of the local Karen community and are fluent in both the Karen language and English. Bicultural workers are critical to supporting community members to understand and apply health-related information within their social and cultural context. The bicultural workers provide BCHS with insights and a deeper understanding of the lived experience of Karen community members. The refugee health nurse supports individuals, families, and refugee communities to improve their health and wellbeing outcomes. BCHS provides refugee settlement services for people from refugee backgrounds from year 1 to year 5 post-arrival. Migrants with low English proficiency are also eligible for settlement support. This includes building health service literacy and safe living for all former refugees. The BCHS is acutely aware that some groups in the community are “hardly reached” meaning that these people are “hardly reached” by the services funded to support them (6). Therefore, the onus is on service providers such as BCHS to consider why their services are hardly reached, and reach out to people/communities, rather than the opposite. Because of the many challenges experienced prior to and during settlement, the BCHS includes former refugee groups within this population of people who may be (or at risk of being) hardly reached.

### 2.2. The Karen community

The BCHS has managed the humanitarian settlement program since 2010. The first Karen family of seven people arrived in Bendigo in 2007. According to Census of Population data, there were 1,597 people who speak Karen at home in the City of Greater Bendigo as of 2021, although the BCHS staff estimate that there are over 4,000 people in the Karen community (7). Prior to settling in Australia, most Karen community members had only known civil war and were stateless. Many had experienced religious and ethnic persecution in Myanmar (8). The Karen community in Bendigo was described as “a very peaceful, passive, hard-working, spiritual community” (P2). BCHS staff also describe the Karen community as “close knit” and that they live communally: *“often [reside in] intergenerational households with multiple family members living together, large families, and they cook a meal that they will have for the whole day.”* (P2). A BCHS bicultural staff member said *“In [a Karen] household we got parent, children, cousin, relative, depend [ing] on how many bedrooms. My family there are eight people in my household now.”* Another BCHS bicultural staff member described the close community ties: *“we always find a way to be in the community setting. For example, those who are Christians, we meet at church, and we always do things together, and everyone know each other. Yeah, and that’s something that I really like about Karen and – yeah, and even in families, we do not want to be separated. We want to be together, so that we can support each other, see each other, and yeah, more of a community rather than being independent and individualizing.” [SIC]* Bendigo welcomes diversity and many of the Karen are positively contributing to the wider community. They are actively employed, attending learning, engaged in sports, and sharing their rich culture with the wider Bendigo community.

Pre-existing inequalities and vulnerabilities rendered the Karen community potentially more at risk from the COVID-19 virus. Some Karen community members are not literate in their own language and have little spoken or written English. One BCHS bicultural staff member explained: “*I think [the] language barrier is the biggest one and there’s also information resource and stuff for the Karen community to know and then because of what their backgrounds, especially in the older generation, with their education [al] background, that they did not have an opportunity to go to school or get an education, so their lack of understanding things – yeah. So even when you explain things to them in a simple way, they still do not get it. They will say that they will get it (to be polite), but they do not understand it necessarily.”* (P9). Language barriers, low levels of formal education, and low literacy in Karen and English created significant health literacy challenges, even with the assistance of interpreters. For example, a bicultural worker explained a situation where misunderstanding may still occur even with an interpreter in a medical consultation: *“the Karen family member might have a low education level in terms of maybe their health information, so they cannot explain to the translator what they mean, and then the translator cannot translate back to them without using medical terms.”* As such, they were less likely to access and understand health advice, less likely to trust health messages shared by government or authority figures, and had low vaccination uptake and close living conditions. Consequently, Karen community members were particularly susceptible to misinformation, such as irrelevant information about the COVID-19 pandemic, or information intended for other communities or settings.

## 3. Community-based program design

The Bendigo Community Health Services recognized that information about health responses to the pandemic shared *via* mainstream mechanisms such as television, radio, and leaflets written in English were unlikely to reach and be effective with Karen community members. These observations and in-practice learnings of BCHS are further supported by research that indicated the Karen community has less access to mainstream news and low levels of health literacy (9, 10). Furthermore, they report lower uptake and use of digital technology than the wider Australian community, and as a former–refugee community, they are located on the “digital fringe” (11). The consequences of the ineffective mainstream media were described by one BCHS staff member: “*So most – a lot of them watching TV, watching social media, and they hear all those things, but like social media or TVs not always true. And sometimes people – usually they just believe whatever they hear, whatever they see, and then they just kind of make up their own scenario…* (P9).” Consequently, the BCHS embarked on a participatory and collaborative approach to identify both barriers and solutions for effective communication strategies. The community-based program design was underpinned by health promotion principles, building on networks of trust, faith-based affiliations, and codesign.

### 3.1. Health promotion based on the Ottawa Charter

The staff at BCHS report that the Ottawa Charter (12) underpins all the work of the Bendigo Community Health Cultural Diversity Team (see Box 1). These health promotion pillars (protection, promotion, and prevention) and strategies (enabling, mediating, and advocacy) drive BCHS’s approach. They also underpin program design and implementation to promote health by addressing the social determinants of health.

Box 1. **The Ottawa Charter.**

Health promotion is the process of enabling people to increase control over and improve their own health. Health promotion is seen as not just the responsibility of the health sector, rather it is the responsibility of all levels of society.

Health is viewed as a resource for everyday life, not the objective of living.

The Ottawa Charter identifies three strategies for health promotion:

1. *Advocate* – health is viewed as a major resource for individuals and community. External political, economic, social, cultural, environmental, behavioral, and biological factors can all favor or harm health outcomes.
2. *Enable* – when focusing on achieving equity in health, a secure foundation in a supportive environment, access to information, life skills and opportunities to make healthy choices are all important factors. People cannot achieve their fullest health potential unless they are able to control the elements that determine their health.
3. *Mediate* – through the coordination of action between all government levels, health, social and economic sectors, non-government and voluntary organizations, local authorities, industry, and the media.

Priority actions for health promotion include developing healthy public policy, creating supportive environments, strengthening community actions, and developing personal skills, thereby enabling people to learn to prepare themselves for all stages of life and if necessary, coping with chronic illness and injuries.

The role of the health sector, therefore, is to move increasingly in health promotion, with responsibility expanding beyond clinical and curative services. Reorienting health services also requires stronger attention to health research, as well as changes in professional education and training.

Reference: World Health Organization. The Ottawa charter for health promotion. Geneva, Switzerland: WHO; 1986 Nov 21 available from: http://www.who.int/healthpromotion/conferences/previous/ottawa/en/index.html.

### 3.2. Building on networks of trust

The Karen community typically learns from and trusts health information that has been shared by networks such as family, friends, and community members, including in their former home country overseas (13). Reliance on these sources of health information was problematic during the height of the pandemic as health information regarding COVID-19 safe behaviors and vaccinations in response to COVID-19 variants was rapidly changing. Furthermore, there was a high likelihood that this health information might be incorrect, misunderstood, or context-specific. For example, there were different regulations in place between the greater Melbourne area and Bendigo, which was classified as a regional setting.

### 3.3. Faith-based affiliations

Within the Bendigo Karen community, there are two primary religious groups, Buddhists and Christians. Because of these close-religious ties within the Karen community, BCHS recognized an opportunity to deliver and share important up-to-date health messages *via* Karen religious leaders. Karen religious community leaders are a trusted source of information, particularly when it is place-based, that is, delivered at home or places of worship such as the local Buddhist temple. The temple is more than a place of worship; it is a safe space, a community center with religious and social significance for local Karen community members. Many community events and festivals are held there. Faith-based affiliations have been increasingly recognized for their potential role in public health, especially during the COVID-19 pandemic (14). One key religious leader was a trusted spokesperson for both groups. Below, he recounts his role in bringing together people from across the two religions under the broader banner of Karen community membership: *“And one thing – important thing, our background is we have two religions: Buddhist and Christian. But when I started, I see it’s a lot of problem, because sometimes some of the people look at me, [and say] ‘I’m Buddhist.’ It’s very hard. But what I do, I never think about the religions. It’s for community.*” (P5)

### 3.4. Codesign

From March 2020 to June 2022, the BCHS refugee team recognized the importance of working with the local Karen community to help ensure COVID-19 health information was easily understood, readable, culturally safe, and relatable. The BCHS refugee team achieved this through a process of codesign described as “active collaboration between stakeholders in designing solutions to a prespecified problem. It promotes citizen participation to formulate or improve specific concerns” (15). In this context, codesign is a participatory approach that brings together BCHS staff and the Karen community to design local solutions to local problems. A BCHS staff member explained: *“It would be absolutely arrogant of us not to codesign. I cannot walk in their shoes*…*You need to embed lived experience in program design and understanding of unmet need.”* (P2). In this way, codesign indicates collective creativity, as it is applied across the whole span of a design process (16), and is further evidence of the influence of the Ottawa Charter (12) on the work of the BCHS as it is a key enablement strategy. Codesign was crucial to develop tailored, targeted, and trusted messages about COVID-19 that would be more likely to reach and be effective with the Karen community (17). However, partnering with people from diverse backgrounds is particularly challenging in remote, rural, and regional areas, despite these communities producing significant innovation (18). Therefore, the BCHS refugee team developed a variety of strategies to deeply engage with the Karen community. For example, codesign sessions provided an opportunity for the community members to ask questions specific to their own needs and enabled staff to dispel misinformation. The bicultural (Karen) staff from the BCHS were integral to this process by facilitating deep connection to the Karen community and sharing their lived experiences which enhanced their understanding of the cultural context and ensured the messages were not lost in translation. By drawing on local wisdom, this process of codesign also identified opportunities to build on activities and networks of trust already implemented and established in the Karen community.

## 4. Community-based program strategies

### 4.1. Codesigned COVID-19 safe messages and the resource hub

The BCHS established a COVID-19 resource hub on its website in March 2020 (19).

The BCHS staff and refugee team (including the bicultural staff) applied the following process to codesigning COVID-19 safe messages:

1. They assessed the suitability and relevance of COVID-19 resources from validated sources such as the Australian Government Department of Health and Human Services.
2. They searched for existing Karen translations and determined whether the translated material was accurate, readable, and relatable. Key criteria for use included whether it was culturally safe, easy to understand, or too dense for local community members.
3. If not translated into the Karen language, or if the translation was unhelpful, two bilingual staff members reviewed and translated the material, focusing on key messaging. The content of the messages was checked by BCHS nursing staff and health professionals.
4. At least two Karen speakers and two Karen readers reviewed the material to ensure accuracy, readability, and relatability. This stage may have included a number of iterations. A BCHS bicultural staff member explained: “*We take information from DHHS, from the government website page, they develop the script and then we translate. From the beginning, I remember they send it to [an independent] translator and then when it’s come back, we have to double check again because some words, it does not make sense.*” (P4)
5. The material was then piloted among BCHS bicultural staff before disseminating to the Karen community.

This five-step process goes beyond translation to consider the community values and norms and frame of reference to support the community to understand and apply the information in their local context (17).

The COVID-19 material included English subtitles to support conversations between Karen and English-speaking/reading staff and community members. Topics initially included: “what is COVID-19,” “social distancing,” “hand hygiene,” and “when you have tested positive to COVID-19.” The focus of the messages changed over time in response to COVID-19 safe directives, for example, topics developed later included building confidence in vaccinations and “how to access and use Rapid Antigen Tests” (self-administered tests for the COVID-19 virus).

Context-specific information resources were also developed for local Karen businesses in response to COVID-19 outbreaks in the local area, such as translated information on advice for close contacts, testing and worker support payments, and COVID-19 safe business practices such as money handling. Information was codesigned in multiple formats (print, audio, and audio–visual) and disseminated across various platforms including over the phone, *via* text message, audio grabs, Facebook, face-to-face, and online information sessions with language support. The codesigned resources were provided to schools, churches, and community groups.

### 4.2. Digital story videos

Codesigning and disseminating digital stories with people from marginalized and diverse groups requires careful consideration of the context in which digital stories are created and where and how they might be shared to ensure an engaged audience response (20). Written material was generated in tandem with over 60 videos in the Karen language. Content featuring photos and videos including trusted community leaders and Karen staff sought to build trust within the local community [see (21)]. The video topics included time and context-specific information to counter misinformation in the community. Digital story topics included lockdown restrictions, mask-wearing, self-care, vaccination and vaccine hub reporting, and a special series of videos to support parents living in isolation with children during the pandemic. The videos were launched online on the Bendigo Coronavirus Refugee Resources Hub. As part of a COVID-19-inspired anti-racism campaign, one of these videos featured the local Mayor, who raised awareness of racism and the reporting of inappropriate behavior after reports of Coronavirus taunts and abuse toward Karen community members. The videos were released on social media sites, Facebook, YouTube, and the BCHS resource hub. The videos had varied numbers of views. The most views were for a video about COVID-19 in the Karen language (775 views). A video where local Karen community members explained why they chose to be vaccinated against COVID-19 (August 2021) had 85 views. A BCHS bicultural staff member reported the value of the videos for community members: “*I think the best way is videos, because a lot of them does not read. And sometimes people – when they look at a big heap, chunk of information, they do not want to spend a lot of time reading them. So, they would prefer videos. So, videos are a really good way for them [to understand the information]*” (P9). [*SIC*]

### 4.3. Telephone hotline operated by BCHS bicultural staff

By July 2020, BCHS had established a virtual network with other service providers in the community specific to the management of COVID-19. Together with this network, BCHS staff conducted community consultations which identified the need for additional telephone support. A free telephone hotline was established for Karen community members in response to the large volume of calls the BCHS bicultural staff were receiving from their community. Many of these calls indicated that the state and federal public health messages were not well understood by the Karen community. The hotline was staffed 5 days a week by bicultural staff, supported by accredited health professionals. Daily briefings were held to ensure that all staff had the most up-to-date information. Advertising of the telephone hotline included word of mouth, online audio–visual media, and dissemination of business cards to local Karen community members and businesses that were trusted by their community.

Many of the calls received by the BCHS bicultural staff were about coronavirus restrictions relevant to local community behaviors, e.g., “Can I go fishing in New South Wales?,” “Can I go to Melbourne?,” isolation, testing, and reporting of positive results. The most common hotline query was about how to obtain financial support from the government through JobKeeper and the Pandemic Leave Disaster Payment or Crisis Payment. The content of the calls to the hotline indicated that some Karen community members had difficulty comprehending the changing rules and restrictions, such as when federal and state restrictions eased. Other difficulties increased when individual community members had to determine how the information applied to their personal decisions, e.g., whether to attend work and whether to send children to school.

The majority of calls to the hotline were for assistance and support with COVID-19 vaccinations. The Karen community experienced many challenges in this regard, including using online booking systems, understanding vaccination information and schedules, and navigating the health system to find vaccination providers. BCHS bicultural staff referred Karen community members to written and video content on the BCHS website, as well as other appropriate sources of information that could be trusted by the community. A BCHS bicultural staff member recounted conversations with local Karen community members: “*we get a call, and they say, “Oh, is that true that… the vaccines, they put the microchip in there..?” (I say) Like, “No, never heard it.” But they say, “Oh yeah, I saw the video.” I’m like, “Where’s the video come from?” (they say) “Video is from America. Video is from [elsewhere].” (I say) “They’re not Australian government. So yeah, you live in Australia, so you should trust the Australian government. And our community. So, we are telling the truth. Not lying. So yeah, you should trust [us].”* (P3)

Approximately 3,120 calls from Karen community members (not all from the Bendigo area) were made to the hotline from June 2020 to June 2022. The number of calls averaged between 30 and 50 calls per week, fluctuating partly in response to changing local, state, and federal directives. The Karen family groups often sat together around the phone to listen to the information provided by the BCHS bicultural staff. The hotline served the Karen community in a range of ways, including effectively delivering COVID-19 safe messages, providing support for people who were isolated, and supporting access to culturally appropriate resources, such as food.

### 4.4. Vaccination clinics in a local Karen temple

The Bendigo Community Health Services piloted an ethno-specific COVID-19 vaccination clinic at the local Karen Buddhist temple. To do this, they leveraged existing community connections, trusted relationships, and the support of the Bendigo Health Covid Outreach Team (who provided a mobile place-based vaccination service) and the Karen Cultural and Social Support Foundation. Further vaccination clinics were held based on the success of the pilot. This initiative relied on bicultural support at each step to ensure the cultural safety of the community. Activities to support community participation prior to the vaccination clinic included: pre-registration *via* the Karen hotline; providing a detailed explanation of the risks, health contraindications, and informed consent prior to the clinics (in Karen language); reminder calls. Bicultural support on the day of the vaccination clinic included assistance with parking, screening, QR code entry, administrative support before, during, and post-vaccination, and follow-up calls.

A BCHS bicultural staff member reported that: “*having a Karen clinic is very helpful for Karen community members. Especially for people who do not understand English. So, for the people who do not understand English, that is really helpful. For me, I feel like when I go there as a client, I feel like much easier to communicate or to go there. So, you do not have to worry about your English. You do not have to worry about how to get the information as well, I think. So that is very useful thing that Bendigo Community Health Services organized (and) created that clinic.*” (P7)

A BCHS staff member observed from the experience of the Karen community attending the vaccination clinic: “*When they get there [the temple], the Karen flags are flying. That’s the first thing. Their flags are so important to them. And because the Monk says it’s [vaccination] a good thing, it’s [vaccination] a good thing. Well, they are booked, they are greeted at the door with myself, usually, and a Karen-speaker. We are very courteous. Then they go through, and I think it’s the familiarity with the language, the trust in us.”* (P2)

The vaccination clinics involved local Karen community member volunteers to help with transportation to and from the venue, parking, crowd management, as well as food and reassurance for others attending for vaccination. This added to the positive experience for the attendees as explained “*And so, when people come – and we also have a volunteer and translator. Everything is organized. When they enter the front [door], they do not need to say anything. Or our volunteer asks them what they need. So very clear for them, and most are very happy. All! I say all are happy! Very well!”* (P5)

To encourage participation, the place and timing of the vaccination clinics were promoted in the COVID-19 safe videos created by BCHS. A BCHS staff member explained: “*every video we did from then on, was ‘book your vaccination’ in Karen language. Sometimes we had waitlists of 70 and 80 [people] … I know that in the metro area they are now talking about the government saying, ‘Let us get out to temples’ … well, we have always done that.”* (P2). Overall, the BCHS and Bendigo Health delivered 17 outreach vaccination clinics at the Karen Buddhist temple. The vaccination clinics were held on the weekends to support community members who work or study during the week. Notably, all faith denominations (both Christian and Buddhist) accessed these vaccination clinics.

## 5. The impact of codesign and community outreach for COVID-19 safe communities

This case study outlines how a regional community health service ensured that information about COVID-19 safe directives from local, state, and federal governments reached the Karen community. The BCHS response was multi-layered, employing a ground-up rather than top-down management approach, and recognized both the strengths and vulnerabilities of the local Karen community. These strengths included a willingness to learn and embrace COVID-19 safe practices, rich cultural traditions, multi-generational familial ties and living arrangements, existing leadership structures, and a strong sense of community. Their vulnerabilities included their previous refugee experience (e.g., trauma, fear of authorities), low (no) proficiency in English, less access to digital technology, and socioeconomic disadvantage. The approach used by BCHS leveraged trusted relationships with local community leaders and champions, including religious leaders who could harmonize with the community. A BCHS staff member explained: “*the Karen community are digitally poor, have poor English, poor literacy in their own language, and are frightened, so [we] had to do it a different way”* (P2). The ground-up approach and engagement with community leaders and bicultural workers helped the BCHS to effectively identify information intermediaries to bridge communication gaps, reduce fear and build trust (22).

A suite of offline/online resources was codesigned including fact sheets, videos, and telephone hotline support. The Karen community favored these resources because social media messaging, text messaging, and telephone calls were previously established and therefore were familiar. Karen community members participated in the codesign process, which had the added benefit of upskilling BCHS staff in understanding and working with the Karen community. The mutual benefits of the codesign process were explained by a BCHS staff member: *“And so authentic codesign is working with people with lived experience and in many ways, I’m just a conduit because you have got the community need, we have identified, then you have got the content expert, problem gambling (for example), or COVID experts … and then you have got the community codesigning in a way that their community will understand what it is and what to do about it*” (P2). This further demonstrates the critical role of bicultural workers as information intermediaries to support the communication of public health information (23, 24).

Interviews conducted with 26 Karen community members at a vaccination clinic found that 77% talked to family and friends when making health-related decisions. Therefore, this ripple effect outward from BCHS to BCHS staff bicultural staff and flowing through to the community was critical to extending the reach of the messages. Similar to other studies, cultural cohesiveness and strong interpersonal relationships helped to drive the communication of COVID-19 health information (25).

This codesign approach supported Karen community members to engage with the suite of materials that were provided in a range of audio–visual formats and hosted on a variety of platforms. BCHS bicultural staff members referred community members to additional material to support decision-making. For example, BCHS bicultural staff members provided verbal information to callers using the hotline. They also provided their community members with printed factsheets and referred them to online videos hosted on accessible platforms (such as the BCHS website and YouTube). Younger, more digitally enabled family members were advised by the BCHS bicultural staff to support older family and community members to access the content. The role of younger people in their communities has been recognized as critical for improving communication networks, especially where their family members do not speak the country’s language (22). The online materials were further supported and promoted by offline materials provided in frequently visited community places and spaces (e.g., Karen grocery store, Karen mechanic, and faith-based places), thereby reinforcing messages in different formats and in places trusted by the community.

The vaccination clinics were supported through hybrid practices including the information provided to hotline callers, promotional videos, phone bookings, and written information. On the day of the vaccination clinic, the community was provided with further support as explained by a BCHS staff member: “*we have interpreters that work with the immunizers so that the consent … [is] informed. We’ve already gone through the consent questions on the phone with them while we do the booking, in language, so they are familiar with the questions they will get asked [at the vaccine clinic].”* (P2). Karen community members were key to managing the process. They helped facilitate car parking, supported visitor flow through the temple (now a vaccination clinic), and provided food and beverages. This strategy had both practical and emotional outcomes. Community members were informed about the process prior to their visit, and they knew what to expect, and when, thereby reducing waiting times. During their visit, they were greeted and supported by familiar and trusted community members and friends, providing added reassurance in language. In these ways, the ethno-specific vaccination clinics (26) respectfully incorporated the lived experience and wisdom of the Karen community to mitigate barriers such as language, culture, communication, mistrust, access, and socioeconomic difficulties.

The codesign approach was facilitated by trusted relationships between the Karen community and BCHS, as well as BCHS and the government. This trust was previously built through the provision of specific services to the Karen community. A BCHS staff member explained: *“People know about our brand, however/whatever the brand is, wherever it is, and there’s trust. So, when we send out some information, either it’s a YouTube (video) with a brand, in language or whatever, they can see it’s from us.”* (P2). Interviews with 26 Karen community members attending a vaccination clinic revealed that 62% of those interviewed accessed information about COVID-19 from BCHS sources and referred specifically to the audio–visual and other materials translated into their language. Other sources of information included Karen community leaders (54%), family (38%), and friends (30%).

A key success factor in these trusted relationships was the role of the leaders as transversal enablers, that is people who actively and intentionally create connections between culturally different residents in their local area—such as the role played by the BCHS staff and Karen community religious leader (27).

The public health approaches described in this case study were enacted on three levels: community, local service providers, and government. It was facilitated by utilizing a combination of approaches that included codesign, place-based, and offline/online flexibility and reinforcement. All of this was founded on a trusted, respectful relationship between BCHS and the local Karen community. This was the key to ensuring COVID-19 safe practices, including vaccination uptake, within this setting.

We recommend the following for community service providers:

- Work closely with local communities and understand their health literacy needs, their strengths, and challenges.
- Actively embed codesign approaches into all new evidence-based health information that does not reach local community members.
- Respond flexibly to changes in circumstances and context, targeting the delivery of information where it will provide the most benefit.
- Encourage the participation of community leaders in delivering health messages online and in person.
- Seek to sensitively embed health messages in local spaces and places where community members reside and meet, including places of worship.
- Ensure flexibility in approaches (e.g., paper, digital, video, telephone hotline) depending on the characteristics of the targeted community.
- Work closely with trusted community leaders and encourage them to participate in codesign and showcasing activities such as cocreating and featuring in context-specific digital stories targeted at their local community members.
- Ensure messaging is provided in the community members’ home language, including the local dialect.
- Ensure bottom-up rather than top-down approaches are used.
- Adhere to the principles of the Ottawa Charter.

## Data availability statement

The original contributions presented in the study are included in the article/supplementary material, further inquiries can be directed to the corresponding author.

## Ethics statement

The research design and procedures were approved by the Swinburne University of Technology (reference: 20215835-8042) and the Peninsula Health Human Research Ethics Committees (reference: HREC/77306/PH-2021). The patients/participants provided their written informed consent to participate in this study.

## Author contributions

HD involved in the literature search, data analysis, and extraction, as well as manuscript preparation. SE involved in data analysis and manuscript preparation and led the research project. KG led the campaign design and implementation, as well as supported interviews, manuscript interpretation, and preparation. CL involved in the literature search, conducted interviews, and supported manuscript preparation. All authors contributed to the manuscript and approved the submitted version.

## Funding

This research project was funded by the Connect Health and Community, Merri Health, Star Health, and Peninsula Health.

## Conflict of interest

The authors declare that the research was conducted in the absence of any commercial or financial relationships that could be construed as a potential conflict of interest.

## Publisher’s note

All claims expressed in this article are solely those of the authors and do not necessarily represent those of their affiliated organizations, or those of the publisher, the editors and the reviewers. Any product that may be evaluated in this article, or claim that may be made by its manufacturer, is not guaranteed or endorsed by the publisher.

## References

[1] Young S. Rituals, reassurance, and compliance: government communication in Australia during the COVID-19 pandemic In: Maarek PJ, editor. Manufacturing government communication on COVID-19. Springer studies in media and political communication. Cham: Springer (2022).

[2] Elmer S Osborne RH Cheng C. Actions to address health literacy and equity in social housing, Victoria. Hawthorn, VIC: Centre for Global Health and Equity, Swinburne University of Technology (2022).

[3] Premiers Office Victoria (2020). Roadmap delivering the national plan. Available at: https://www.premier.vic.gov.au/sites/default/files/2021-09/210919%20-%20Roadmap-Delivering%20the%20National%20Plan.pdf (Accessed October 20, 2022).

[4] Gillespie JA Buchanan J Schneider CH Paolucci F. COVID-19 vaccines and the Australian health care state. Health Policy Technol. (2022) 11:100607. doi: 10.1016/j.hlpt.2022.100607, PMID: 35190790 PMC8842088

[5] Shergold P. Broadbent J. Marshall I. Varghese P. Fault lines: An independent review into Australia’s response to COVID-19. (2022). Downloaded from: https://www.paulramsayfoundation.org.au/news-resources/fault-lines-an-independent-review-into-australias-response-to-covid-19 (Accessed February 14, 2023).

[6] Sokol R Fisher E Hill J. Identifying those whom health promotion hardly reaches: a systematic review. Eval Health Prof. (2015) 38:518–37. doi: 10.1177/0163278715605883, PMID: 26405265

[7] Australian Bureau of Statistics, Census of Population and Housing (2021). Available at: https://abs.gov.au/census/find-census-data/quickstats/2021/LGA22620 (Accessed October 26, 2021).

[8] Deloitte Economics. Economic and social impact of the Karen resettlement in Bendigo, A Joint Ames Australia and Deloitte Access Economics Report. (2018). Available at: https://www2.deloitte.com/content/dam/Deloitte/au/Documents/Economics/deloitte-au-economics-regional-futures-karen-resettlement-ames-031018.pdf

[9] Griffin G Nau SZ Ali M Riggs E Dantas JAR. Seeking health information: a qualitative study of the experiences of women of refugee background from Myanmar in Perth, Western Australia. Int J Environ Res Public Health. (2022) 19:3289. doi: 10.3390/ijerph19063289, PMID: 35328976 PMC8951186

[10] Wong CK White C Thay B Lassemillante ACM. Living a healthy life in Australia: exploring influences on health for refugees from Myanmar. Int J Environ Res Public Health. (2020) 17:121. doi: 10.3390/ijerph17010121 PMC6982124 31877976

[11] Hespanhol L Davis H Fredericks J Caldwell G Hoggenmüller M Farmer J. The digital fringe and social participation through interaction design (introduction to special issue). J Community Inform. (2018) 14:4–16. doi: 10.15353/joci.v14i1.3399

[12] World Health Organisation. (1986). Ottawa charter for health promotion: First international conference on health promotion Ottawa, 21 November, 1986. Available at: https://www.healthpromotion.org.au/images/ottawa_charter_hp.pdf

[13] Lloyd A. Building information resilience: how do resettling refugees connect with health information in regional landscapes–implications for health literacy. Australian Acad Res Libraries. (2014) 45:48–66. doi: 10.1080/00048623.2014.884916

[14] Tomalin E Sadgrove J Summers R. Health, faith and therapeutic landscapes: places of worship as black, Asian and minority ethnic (BAME) public health settings in the United Kingdom. Soc Sci Med. (2019) 230:57–65. doi: 10.1016/j.socscimed.2019.03.006, PMID: 30965184

[15] Vargas C Whelan J Brimblecombe J Allender S. Co-creation, co-design and co-production for public health: a perspective on definitions and distinctions. Public Health Res Pract. (2022) 32:e3222211. doi: 10.17061/phrp3222211, PMID: 35702744

[16] Sanders E Stappers P. Co-creation and the new landscapes of design. CoDesign. (2018) 4:5–18. doi: 10.1080/15710880701875068

[17] Wild A Kunstler B Goodwin D Skouteris H Zhang L. Communicating COVID-19 health information to culturally and linguistically diverse (CALD) communities: the importance of partnership, co-design, and Behavioural and implementation science. MetaArXiv. (2020). doi: 10.17061/phrp2020-0094

[18] Davis H Farmer J. Including the rural excluded: digital technology and diverse community participation In: Dezuanni M Foth M Mallan K Hughes H, editors. Digital participation through social living labs: Valuing local knowledge, enhancing engagement Cambridge, UK: Chandos Publishing (2017). 223–44.

[19] Karen COVID-19 Resources. Bendigo Community Health Services Karen COVID-19 Resource Hub. Available at: https://www.bchs.com.au/blog/881-karen-covid-19-resources (Accessed October 1, 2022).

[20] Davis H Waycott J Schleser M. Digital storytelling: designing, developing and delivering with diverse communities (in) In: Miettinen S Sarantou M, editors. Managing complexity and creating innovation through design. London: Routledge (2019)

[21] Archived Karen COVID-19 Resources. Bendigo Community Health Services Archived Karen COVID-19 resources published in 2020 and 2021. Available at: https://www.bchs.com.au/blog/882-archived-karen-covid-19-resources (Accessed October 1, 2022).

[22] Seale H Harris-Roxas B Heywood A Abdi I Mahimbo A Chauhan A . The role of community leaders and other information intermediaries during the COVID-19 pandemic: insights from the multicultural sector in Australia. Human Soc Sci Commun. (2022) 9:1–7. doi: 10.1057/s41599-022-01196-3

[23] Ioannidesa SJ Hessb I Lambertond C Luisib B Guptab L. Engaging with culturally and linguistically diverse communities during a COVID-19 outbreak: a NSW Health interagency public health campaign. Strategies. (2022) 5:8–10. doi: 10.17061/phrp32342215 36315850

[24] Carlson SJ Edwards G Blyth CC Nattabi B Attwell K. 'Corona is coming': COVID-19 vaccination perspectives and experiences amongst culturally and linguistically diverse west Australians. Health Expect. (2022) 25:3062–72. doi: 10.1111/hex.13613, PMID: 36262050 PMC9700143

[25] Healey SJR Ghafournia N Massey PD Andrich K Harrison J Taylor K . Factors contributing to the sharing of COVID-19 health information amongst refugee communities in a regional area of Australia: A qualitative study. BMC Public Health. (2022) 22:1–11. doi: 10.1186/s12889-022-13850-1 35897090 PMC9331021

[26] Stadnick NA Cain KL Oswald W Watson P Ibarra M Lagoc R . Co-creating a theory of change to advance COVID-19 testing and vaccine uptake in underserved communities. Health Serv Res. (2022) 57:149–57. doi: 10.1111/1475-6773.13910, PMID: 35243622 PMC9108217

[27] Wilding R Nunn C. Non-metropolitan productions of multiculturalism: refugee settlement in rural Australia. Ethn Racial Stud. (2018) 41:2542–60. doi: 10.1080/01419870.2017.1394479

